# Case study of IMU loads and self-reported fatigue monitoring of water polo goalkeepers preparing for the Olympic games

**DOI:** 10.3389/fspor.2023.1198003

**Published:** 2023-05-15

**Authors:** Félix Croteau, Sylvain Gaudet, Jeremy Briand, Julien Clément

**Affiliations:** ^1^ Institut national du sport du Québec, Montréal, QC, Canada; ^2^ Water Polo Canada, Montréal, QC, Canada; ^3^ School of Physical and Occupational Therapy, McGill University, Montréal, QC, Canada; ^4^ Département de Génie des Systèmes, École de Technologie Supérieure, Montréal, QC, Canada

**Keywords:** aquatic, fatigue, inertial measurement unit, monitoring, water polo players

## Abstract

**Introduction:**

Measurement of training in water polo goalkeepers has focused first on psycho-physiological variables, but also on external volume estimated with wearable sensors. However, there are limited studies exploring training monitoring in water polo goalkeepers longitudinally.

**Methods:**

Three female senior national team goalkeepers participated in this study from May to August 2021. Internal loads were defined using session rating of perceived exertion (sRPE). Tri-axial accelerations and angular velocities were measured with an inertial measurement unit (IMU) placed on the lower back to measure external loads. Relationships between self-reported and IMU-derived metrics were explored using Spearman correlations. Two-way ANOVAs were used to assess differences between session types and between athletes.

**Results:**

In total, 247 sessions were collected (159 practices, 67 matches and 21 game warm up), with 155 sessions having complete data. IMU metrics, such as number of kicks, number of jumps or player-load showed high correlation with each other (*ρ* = 0.80–0.88). There was also a moderate correlation (*ρ* = 0.47, 95% CI = 0.33–0.58) between sRPE and player-load measured with the IMU. ANOVA tests showed that there were significant differences between athletes for sRPE (*p* < 0.01) but not for player load (*p* = 0.47). There were no interactions between athletes and training types, except for index score (*p* < 0.01).

**Conclusions:**

This study shows that monitoring of training loads can be performed successfully in water polo goalkeepers using a combination of self-reported and IMU measures. Self-reported outcomes can be expected to vary significantly across athletes within the same session, while IMU metrics vary across training situations. Finally, coaches should be mindful of missing data, as they can skew the interpretation of training loads.

## Introduction

Water polo is an aquatic team sport where participants must swim across a 25 m (for women) or 30 m (for men) pool and shoot a ball into the opposing team's net (1). This requires multiple bouts of sprinting throughout the match, as well as periods of static play where players must stay upright in the water. During this time, goalkeepers must continuously tread water between each bout where their team is in a defensive stance (2). To block the ball, the goalkeepers must perform sliding actions across the water to cover the horizontal space in the net (3 m wide), as well as jump to reach for balls thrown in the upper areas of the net (0.9 m tall) (3). Furthermore, they must often keep their arms out of the water to be ready to block the ball, increasing the task difficulty of treading water without support (4). This range of skills can be trained in different manners, for which the readers are referred to more specific publications available on the topic of water polo physical preparation (5, 6). However, much less literature is available on the monitoring methods available to track water polo goalkeepers' training loads (7). While other team sports have embraced global positioning systems (GPS) and heart rate (HR) monitoring technologies to track training over time, water polo provides a unique challenge for using electronic devices given its partaking in an aquatic environment (often indoors) (8). Athlete monitoring, or the ability to carefully observe and document training and its effects on players, is paramount for coaching staff to optimize the favorable adaptations expected from training. This can serve to identify periods in the training cycle where athletes are ready for higher bouts of intensity or volume, or when they would benefit from additional rest and recovery (9). By optimizing the management of their training status, coaches can help minimize negative adaptations such as overuse injuries (10, 11).

Most publications so far have explored psycho-physiological variables of training in water polo. Some authors have explored means to document the subjective effects of specific training regimes via questionnaires about perceived fatigue (12, 13), or by calculating an index of heart rate values derived from a wearable heart rate sensor (14). Further cross-sectional studies have also investigated the changes to heart rate variability associated with water polo training (15). All of these approaches showed relevance to quantify training load for goalkeepers as well as field players.

Recently, a group of researchers conducted a qualitative study to explore the end-user benefits of documenting session rate of perceived exertion (obtained by multiplying the session duration by the rating of perceived exertion, referred to as sRPE throughout this manuscript) in water polo players (16). They argue that although this approach provided insights when athletes were not adapting to the training goals, it failed to inform coaches about what training content led to maladaptation. Consequently, the authors conclude that further work is necessary to develop methods that can serve to also quantify training volume and intensity in water polo in terms of swimming and shooting. Preliminary work has shown the potential for wearable inertial measurement units (IMU) to accomplish this goal (7, 17). Therefore, the purpose of this case report is to examine the outcomes of a monitoring strategy implemented for water polo goalkeepers using tools to document perceived exertion from training as well as estimates of training volume or intensity extracted from wearable IMU. We hypothesize that these descriptors of internal and external loads will correlate to a moderate extent only. Next, we submit that each athlete will demonstrate an individual profile of how these variables fluctuate over time.

## Materials and methods

### Participants

Three female goalkeepers from the Canadian senior women's national water polo team took part in this study. They encompass 100% of the goalkeepers in the team. Participants' ages ranged between 23 and 27 years old and height ranged between 172 cm and 179 cm. All three had greater than five years of experience competing at the senior international level. Participants were first provided with information about the study and consented to participate. The experimental protocol was reviewed and approved by the McGill university IRB (study 01-M01-20A) in alignment with the Declaration of Helsinki.

### Data collection

Internal loads were defined as session rating of perceived exertion (sRPE) by multiplying rating of perceived exertion (RPE, 0–10 scale) by the session duration in minutes (18). Data were collected daily after each training session (including water polo sessions, dryland sessions and combined water polo and dryland sessions) between May and August 2021, as part of the ongoing training load monitoring process of the Canadian senior women's national water polo team. The athletes logged their sRPE via an online platform available through their smartphones (Hexfit®, Canada). Afterwards, entries were validated retrospectively by a staff member to identify aberrant durations or incorrect labelling of the training type. Only the sRPE data for water polo and combined sessions were used in the following analysis because IMU data were not collected during dryland sessions.

Tri-axial accelerations and angular velocities were measured with an IMU (Xsens Dot, Xsens Technologies, Enschede, Netherlands) placed on the lower back by a staff member prior to water polo sessions. The IMU signals were filtered and analyzed with a custom Matlab® script (MathWorks, Natick, USA) to create training volume metrics according to previous methods from Clément et al. (7). These included overall player-load (summation of change of accelerations) (19), high-intensity activity counts such as number of jumps and kicks, as well as an index score based on the weighted time spent in different intensity zones. The score is weighed so that a value of 100% would imply that 100% of the session was spent at high intensity.

During the same period, the participants also recorded their pre-training resting HR using a Polar™ H10 chest strap connected to the Polar Beat™ application for smartphones via Bluetooth (Polar Electro, Finland). The participants further used a PUSH Pro Band 2.0 accelerometer connected to the PUSH™ application for smartphones via Bluetooth (Whoop Unite, Boston, USA) to record the peak velocities and estimate jump height during a dryland bout of three counter-movement jumps. These heart rate and jump measurements were collected and included in the same database as sRPE and IMU-metrics but were not included in the analysis due to the excessive missing data (see Discussion).

### Statistical analysis

Descriptive statistics characterize the range of values obtained for each of the variables under study. Statistical tests were performed to convey the direction of inference of the data, and 95% confidence intervals express the limitations in generalizability of this case study (20). Because of the non-normality of the data, relationships between sRPE and IMU-derived metrics were explored using Spearman's rank correlations, with values of 0–0.39 interpreted as weak, 0.4–0.69 as moderate, 0.7–0.89 as strong, and 0.9–1.0 as very strong associations (21). To assess the effect of athlete and session type on the measurements, we conducted a two-way ANOVA (athlete x session type) on the rank-transformed data of each self-reported and IMU metrics and performed Wilcoxon signed-rank tests in post-hoc analysis (22). Multivariate imputation by chained equations approach (MICE) (23) was applied to account for missing sRPE data as it is both flexible and practical to this data scenario because the dataset for IMU variables is nearly complete (24). MICE parameters were set to five multiple imputations, 50 iterations and predictive mean matching (pmm) as the imputation method; the average of the five imputations was retained as the imputed dataset. The imputed dataset was used to explore the temporal changes in player-load and sRPE. Specifically, weekly sums for sRPE and player-load were compared using the original data and the imputed dataset. The complete statistical analysis was conducted in R (25) with *stats, DescTools* (26) and *mice* (27) packages, whereas figures were generated with the package *ggpubr* (28). Statistical tests were computed using a significance level of *α *= 0.05.

## Results

**Figure 1:**
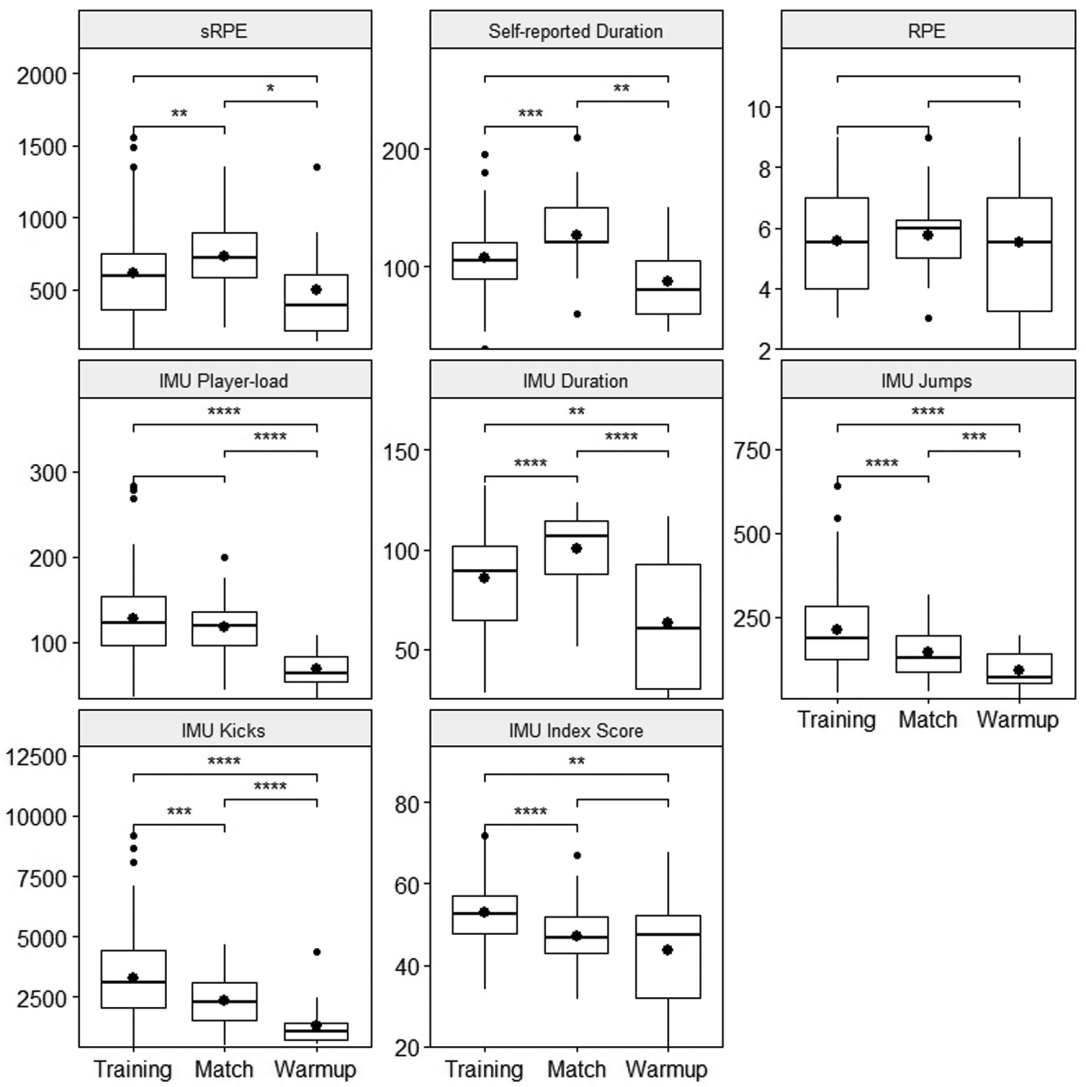
Boxplots showing data distribution for each metric across the three goalkeepers (A1, A2, A3). The dot inside each box indicates the mean. Significant differences based on post-hoc analyses are expressed as follows: *=*p* < 0.05, **=*p* < 0.01, ***=*p* < 0.001, ****=*p* < 0.0001.

**Figure 2:**
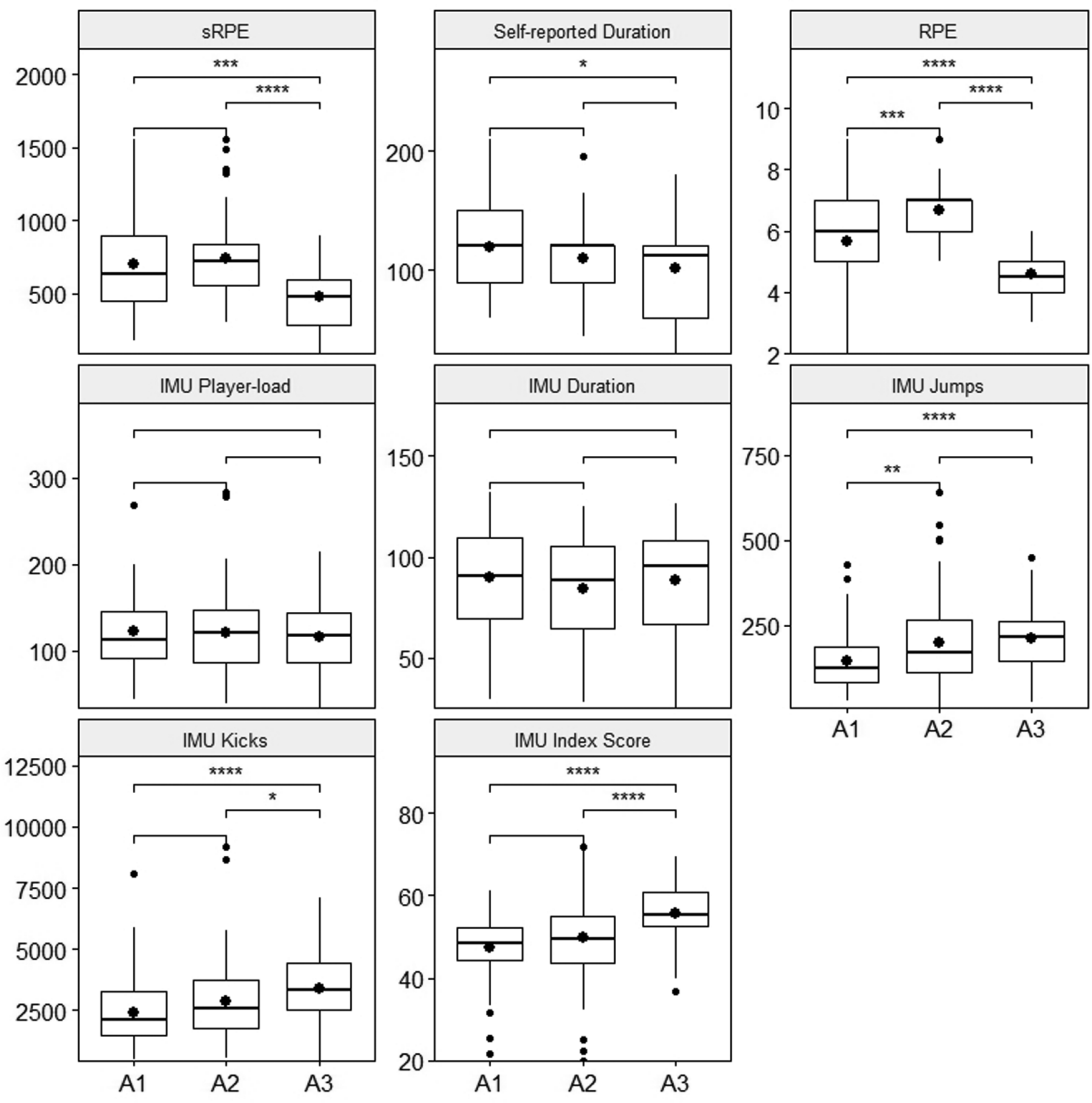
Boxplots showing data distribution of each metric across the three session types. The dot inside each box indicates the mean. Significant differences based on post-hoc analyses are expressed as follows: *=*p* < 0.05, **=*p* < 0.01, ***=*p* < 0.001, ****=*p* < 0.0001.

In total, 247 water polo sessions were observed (159 practices, 67 matches and 21 game warm up), with 155 sessions having complete data. The largest missing data category was sRPE (32%) while IMU recordings were missing for only 5% of sessions. Figures 1,2 present the distribution of each metric relative to athletes and session type, respectively.

IMU metrics such as number of kicks, number of jumps or player-load showed strong correlation with each other (*ρ* = 0.80–0.88, see Table 1 for complete list of correlation coefficients with 95% CI). Self-reported metrics of RPE and sRPE showed strong correlation with each other (*ρ* = 0.70, 95% CI = 0.62–0.77). However, session duration and RPE showed only weak correlation (*ρ* = 0.30, 95% CI = 0.16–0.44). Comparisons were further evaluated between IMU variables and self-reported variables. Of interest, self-reported duration was moderately correlated with IMU duration (*ρ* = 0.63, 95% CI = 0.52–0.71) while sRPE was also moderately correlated with player-load (*ρ* = 0.47, 95% CI = 0.33–0.58).

**Table 1:** Spearman rank correlation coefficients and 95% confidence intervals between internal (self-reported) and external (measured using IMU) training metrics.

	sRPE	Self-reported duration	RPE	IMU player-load	IMU duration	IMU jumps	IMU kicks	IMU index score
sRPE	1.00	0.87 (0.83,0.90)	0.70 (0.62,0.77)	0.47 (0.33,0.58)	0.55 (0.43,0.65)	0.24 (0.03,0.39)	0.26 (0.10,0.40)	−0.20 (−0.35,−0.05)
Self-reported Duration	…	1.00	0.30 (0.16,0.44)	0.42 (0.28,0.54)	0.63 (0.52,0.71)	0.16 (0.00,0.31)	0.23 (0.07,0.37)	−0.30 (−0.44,−0.15)
RPE	…	…	1.00	0.35 (0.20,0.48)	0.22 (0.06,0.36)	0.25 (0.09,0.39)	0.20 (0.04,0.35)	0.01 (−0.14,0.17)
IMU Player-load	…	…	…	1.00	0.72 (0.65,0.78)	0.80 (0.75–0.84)	0.87 (0.83,0.90)	0.38 (0.26,0.48)
IMU Duration	…	…	…	…	1.00	0.45 (0.34,0.54)	0.54 (0.44,0.62)	−0.13 (−0.25,0.00)
IMU Jumps	…	…	…	…	…	1.00	0.88 (0.85,0.91)	0.60 (0.51,0.67)
IMU Kicks	…	…	…	…	…	…	1.00	0.58 (0.49,0.66)
IMU Index Score	…	…	…	…	…	…	…	1.00

The two-way ANOVA analysis revealed that all self-reported ratings of perceived exertion were affected by athlete only (*F* = 36.62, *p* < 0.001), while IMU metrics such as player-load (*F* = 19.88, *p* < 0.001)) and duration (*F* = 15.93, *p* < 0.001) were only affected by session type (Table 2). In addition, sRPE, self-reported duration, number of jumps, number of kicks and index score of the sessions were affected by both athlete (*p* < 0.05) and session type (*p* < 0.01), but only the index score (*F* = 3.36, *p* = 0.01) showed significant interaction between the two factors.

**Table 2:** Two-way ANOVA summary reporting the effect of athlete and session type on training metrics as well as interactions between factors.

	Athlete *F*-value (*p*-value)	Session type *F*-value (*p*-value)	Athlete x session type *F*-value (*p*-value)
sRPE	12.02 (<0.001)	6.72 (<0.01)	0.75 (0.56)
Self-reported Duration	3.20 (0.04)	8.59 (<0.001)	0.33 (0.86)
RPE	37.62 (<0.001)	0.48 (0.62)	1.01 (0.40)
IMU Player-load	0.21 (0.81)	19.88 (<0.001)	1.06 (0.38)
IMU Duration	0.99 (0.38)	15.925 (<0.001)	1.38 (0.24)
IMU Jumps	13.59 (<0.001)	24.63 (<0.001)	1.44 (0.22)
IMU Kicks	11.44 (<0.001)	28.26 (<0.001)	0.32 (0.86)
IMU Index Score	24.67 (<0.001)	17.01 (<0.001)	3.36 (0.01)

**Figure 3:**
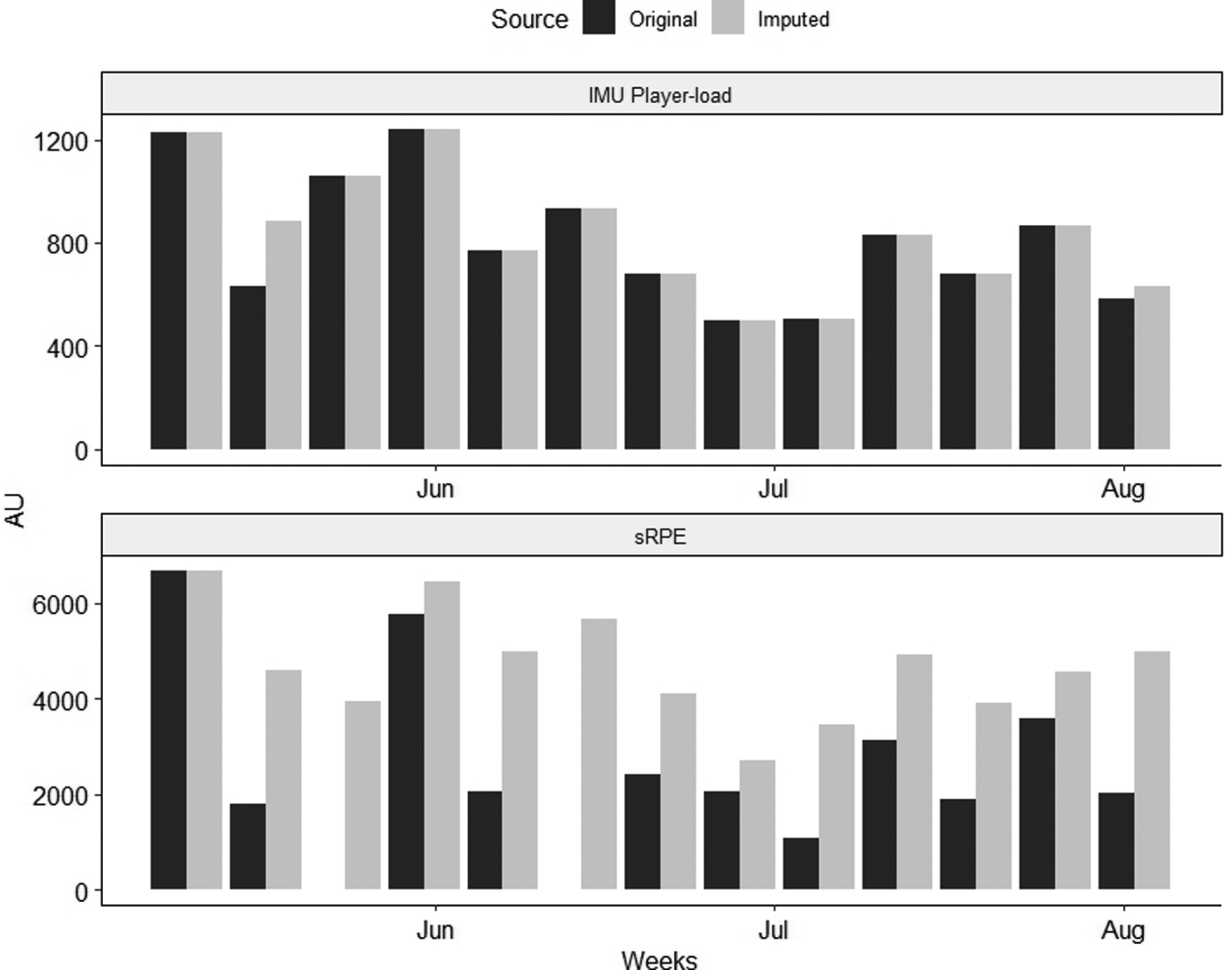
Weekly sums of player-load (IMU) and sRPE (self-reported) for one goalkeeper. Player-load remains similar in both raw and imputed weeks, however large differences are present for weekly sums of sRPE during most weeks. AU, Aabitrary units.

Finally, player-load and sRPE weekly sums for original and imputed datasets of one athlete are presented in Figure 3. Although no statistical analysis was performed here, the higher proportion of missing data for sRPE (32% across the three goalkeepers) compared to player-load (5%) is reflected in this figure where sRPE weekly sums are often underestimated with the original dataset.

## Discussion

The objective of this case report was to demonstrate how measures of perceived exertion and IMU metrics can inform coaches on the adaptations to the prescribed training. Results from the correlation analysis highlight the importance of measuring both types of metrics (internal and external load), as they cannot be considered equivalent, and they provide complementary information about the athletes' training responses. The results of this study also show that self-perceived and IMU metrics are influenced by individual participant, session type, or both.

As reported in Table 1, a moderate positive correlation exists between external load and internal load, namely between player-load and sRPE (*ρ* = 0.47, 95% CI = 0.33–0.58). Furthermore, a weak negative association between sRPE and the IMU index score was found, with a *ρ* of −0.20 (95% CI = −0.35 to −0.05). This implies that the index score decreases as the sRPE increases. We would rather expect a positive correlation between those two metrics, reflecting the energetic cost of performing actions at higher intensity (29). Calculating an index that multiplies the absolute minutes spent in each intensity zone by an incremental factor may correlate more closely with sRPE compared with the current index which is normalized over time (30). Nevertheless, correlation analysis may be an overly simplistic approach to explore the relationship between these concepts. For example, Bartlett et al. (31) found stronger associations between internal and external loads using machine learning modelling (31), however they concluded that individualized models were nonetheless more accurate than group-based models. This may be attributed to the temporal relationship between exposure and adaptation in sports performances (32). As athletes become fitter, the same amount of external load should be expected to require less physiological stress to accomplish (33). While part of the unexplained variance between these two metrics can be rationalized by the individual differences in self-perception of exertion (from ANOVA analysis, discussed later), methodological considerations on how those metrics were collected likely have an effect. Indeed, athletes sometimes performed dryland sessions right before pool training time, which in turn increases sRPE, whereas IMU duration always included only in-water training.

ANOVA analysis showed that certain metrics, both self-reported and measured with IMU, were different between athletes (Table 2). However, the RPE was the metric showing most variability between athletes (Figure 1). Indeed, post-hoc analysis revealed significant differences in RPE scores between each pair of athletes (*p* < 0.01), which in turn lead to significant differences in sRPE except between A1 and A2 (*p* = 0.49). Athlete A3 reported RPE consistently lower than their peers; athlete A2 mainly rated sessions as moderate to hard; finally, athlete A1 reported RPE across the whole scale of values available. These individual tendencies have been observed in previous athlete cohorts as well (34), and could be explained by environmental factors, accumulated fatigue that propagates from day to day or even athletes' interpretation of the scale (35). In contrast, IMU player-load showed no significant differences across participants (*p* = 0.81). This is consistent with the fact that player-load is a measure of external load and is therefore independent of athlete's perception. Furthermore, knowing that all three athletes train on the same team and are all goalkeepers, it is not surprising to observe that training volume was similar between them. Conversely, ANOVA and post-hoc analysis indicated significant differences across athletes for number of kicks and jumps as well as index scores (*p* < 0.001). Altogether, our findings show that although participants performed a similar total volume of training (player-load), it was perceived and executed at different intensities across all three goalkeepers.

Our results showed no significant effect of session type on the ratings of perceived exertion (*p* = 0.62) (Figure 2). However, session type influenced all IMU metrics (player-load, jumps, kicks, index score, IMU duration) and nearly all pairs of session type comparisons (training/match, match/warmup, training/warmup) showed significant difference in post-hoc analysis. Overall, IMU metrics measured in training had the highest values, followed by match, then match warmup, except for the duration which was highest for matches. In contrast, Clément et al. (7) had previously found higher IMU metrics in matches compared with training (7). However, their sample included only seven matches and seven practices, and hence this difference may be due to under-sampling. The fact that ratings of perceived exertion remained unchanged across session types despite external load metrics decreasing could reflect the difference in cognitive load experienced during high-stakes competitions vs. training (13). Indeed, multiple factors are involved in the pathway from external load applied to internal load perceived, acting as a complex system where individual parameters and IMU metrics are influenced by many contextual variables, including the outcome of a competition (36, 37). Altogether, these can impose fatigue that is not only physical, but also mental and emotional (38). On the other hand, it is also possible that the higher proportion of missing values for self-perceived metrics may hide an effect of session type, thus further research is necessary to explore how these variables influence each other in water polo players.

The distribution of self-report and IMU variables illustrates that all three participants perform similar total amounts of movement (player-load) throughout the sessions. However, the index score, which illustrates the portion of time spent at high intensity levels for each session, is the only variable that yields a significant interaction effect between athletes and training type (*p* = 0.01). This may reflect distinct levels of physiological adaptations to those tasks, with certain participants being of greater fitness levels (33). Alternatively, this may instead illustrate that certain athletes perform specific session types with different intensities (i.e., greater intensity in match warm-up). Future research should attempt to develop a weighed score that is not normalized over time, unlike the index presented here. This may show greater sensitivity to intensity differences within sessions and emphasize individual profiles over time (39).

Recently, Bache-Mathiesen et al. (24) have stressed the importance of considering the impact of missing data on statistical inference (24, 40). Knowing that adherence to a monitoring program is always challenging, the last objective of this study was to explore how weekly total sums of internal and external loads could be affected by missing data (Figure 3). Hence, a MICE approach was selected for this application given the low missing data proportion from the IMU measurements and their expected relationship, to some extent, with the RPE ratings (41). In this study, IMU data were missing for only 5% of sessions, hence there are only small differences observed between the original and imputed player-load data. However, the lower part of Figure 3 illustrates the sRPE with and without imputed data. Throughout the entire observation period, there are often crucial differences between the original and imputed data for sRPE. Weeks three and six, for instance, were missing sRPE data altogether for this participant. Therefore, the imputed data allow for a better estimate of the weekly internal load from the player-load values. The interpretation of the change in internal load over time, if only based on original data, may even at times suggest that the direction of change was opposite to what was taking place. This can have severe consequences on preparation for major water polo competitions such as the Olympics, as shown by Botonis & Toubekis (42). Indeed, they showed that increased training load can affect sleep quality negatively, and risk poor athletic performance. Coaches must adapt training prescription to the competition calendar to maximize the odds of peaking at target events (43). Overall, this figure conveys that sport scientists should be cautious when making training plans based on training load data that is incomplete (40).

As recommended by Borg et al. (44), reporting the sources of missing data can help identify challenges of data collection in team sports (44). The missing data from the IMU stems from technical errors, where the devices accidentally stopped recording (i.e., holding on/off button by mistake), or because data transfer was not handled properly. The size of the files resulting from measuring sessions at 30 Hz for 60–120 min requires regular clearing of the IMUs' internal memory, otherwise new recordings were cut short. In contrast with the IMU data, self-reported, physiological (resting HR) and neuromuscular (counter-movement jump) measurements required daily active and autonomous engagement from the participants for an extended period. Unfortunately, nearly one third of self-report data into Hexfit™ were missing by the end of the observation period, which is near the 30% threshold to use mean imputation suggested by Bache-Mathiesen et al. (24). The resting HR data and the PUSH™ jumping data were absent for 43% and 55% of the sessions, respectively. The larger proportion of missing data from the HR monitoring and the PUSH™ devices suggests that making participants enter data on multiple platforms increases the risk of missing data, which was compounded by a lower frequency of measurement (every two days).

Different strategies were used to maximize athletes' adherence with monitoring (as opposed to passive compliance with what is requested or imposed by the team). The experimental process was explained to the athletes ahead of time, and they were provided with periodic feedback about their completion rates. However, adherence could be improved if the athletes had direct access to their information through visualization tools available on a smartphone application (45). This process could ease reflections and conversations between the athletes and the coaches to better understand how daily data monitoring influence training choices. This is particularly important in water polo goalkeepers, where the number of athletes is small and the ability for coaches to adjust their training programs is less time-consuming than for the rest of the team (46). Future studies should implement an adherence strategy from the outset of the study and confirm that the proposal matches the athletes' needs.

### Limitations

Goalkeepers only represent a small fraction of the total number of players within team sports, and as such, players in this position are not commonly the topic of scientific research. The small sample sizes available impose restrictions on the statistical analyses possible, which reduces the generalizability of the findings to other goalkeepers. Therefore, the data were presented visually along with confidence intervals throughout this study for the readers to appreciate the level of uncertainty remaining after the analyses (47). Next, the large amount of missing data highlight the challenges with athletes' adherence in longitudinal monitoring. Data from resting HR and the CMJ values were removed from the analysis altogether because nearly 50% of the data were missing for these metrics, making the inference inaccurate on this data (24). Finally, the MICE approach assumes that data is missing at random and that the observations are normally distributed. The dataset was not entirely normal, mainly skewed by a small amount of outlier values. This is attributed mainly to the small dataset included in this case study (*n* = 3). Moreover, the possibility that missing data was not completely random still exists, but further measurement on contextual factors would be necessary to confirm this hypothesis. The absence of randomness would indeed skew the methods for the MICE approach.

## Conclusion

Monitoring of training load can be performed in water polo goalkeepers using a combination of self-reported and IMU measures to inform coaching decisions. Self-reported outcomes can vary significantly across athletes within the same session. IMU volume data are sensitive to session type but less affected by individual athletes (unlike intensity measures), therefore they should be included to give further context for coaches. Our findings also suggest that a weighed measure of training that considers intensity levels may ultimately be superior to understanding individual player profiles. Finally, missing data can skew the interpretation of training loads. A strategy should be implemented to first obtain reliable data from the outset, but also to account for missing data before making conclusions.

## Data Availability

The datasets presented in this article are not readily available because Participants did not provide consent to secondary use of the dataset. Requests to access the datasets should be directed to fcroteau@insquebec.org.
